# Comparison of Intravesical Bacillus Calmette-Guérin (BCG) and Mitomycin C for the Treatment of Non-Muscle-Invasive Bladder Cancer

**DOI:** 10.7759/cureus.86524

**Published:** 2025-06-22

**Authors:** Kamran A Shah, Syed Rafiuddin Shah, Waqas Ahmed Khan, Sara Nisar, Syeda Farwa Zaidi, Muhammad Asif, Farah Gul, Farid Ullah

**Affiliations:** 1 General Surgery, University Hospitals Birmingham NHS Foundation Trust, Birmingham, GBR; 2 Urology, Sindh Institute of Urology and Transplantation, Karachi, PAK; 3 Oncology, Aga Khan University Hospital, Karachi, PAK; 4 Internal Medicine, Women Medical and Dental College, Abbottabad, PAK; 5 Internal Medicine, Rehman Medical Institute, Peshawar, PAK; 6 Urology, DHQ Teaching Hospital, Mardan, PAK; 7 Pharmacology, Pakistan Council of Scientific and Industrial Research, Peshawar, PAK; 8 Medicine, Khyber Teaching Hospital, Peshawar, PAK

**Keywords:** bacillus calmette-guérin, disease progression, intravesical therapy, mitomycin c, nmibc, recurrence

## Abstract

Background

Bacillus Calmette-Guérin (BCG) and mitomycin C (MMC) are the two most commonly used intravesical therapies for non-muscle-invasive bladder cancer (NMIBC), yet variability in treatment outcomes and tolerability continues to challenge clinical decision-making. Updated comparisons reflecting current treatment protocols and adherence patterns are needed to inform practice.

Objective

The objective of this study is to evaluate and compare the efficacy and safety of intravesical BCG versus MMC in the contemporary treatment of NMIBC, focusing on recurrence, progression, and adverse effects over a 24-month follow-up.

Methods

This comparative study was conducted at the Khyber Teaching Hospital over a two-year period from January 2023 to December 2024. A total of 286 patients were enrolled, with 143 patients in each treatment group. Patients who had a complete transurethral resection of bladder tumor, were confirmed to have NMIBC, were at least 18 years old, and were eligible for intravesical therapy with either BCG or MMC were included in the study. Patients with muscle-invasive bladder cancer, BCG or MMC contraindications, active UTIs, severe immunosuppression, or serious comorbid diseases that might affect therapy results were excluded.

Results

The BCG group had much lower rates of cancer returning after 12 months (19 out of 143 patients, 13.29%, vs. 32 out of 143 patients, 22.38%; p = 0.037) and 24 months (34 out of 143 patients, 23.78%, vs. 49 out of 143 patients, 34.27%; p = 0.043), a longer average time before cancer returned (14.20 ± 4.80 months vs. 10.90 ± 5.30 months; p = 0.026), and less disease progression after 24 months (15 out of 143 patients, 10.49%, vs. 29 out of 143 patients, 20.28%; p = 0.021). Adverse effects were more frequent in the BCG group (n = 67, 46.85% vs. n = 58, 40.55%; p = 0.312), with fever (n = 19, 13.28% vs. n = 6, 4.19%; p = 0.012) and flu-like symptoms (n = 24, 16.78% vs. n = 8, 5.59%; p = 0.004) occurring more commonly.

Conclusions

BCG demonstrated superior efficacy over MMC in reducing recurrence and progression rates in patients with NMIBC, with a higher frequency of systemic adverse effects.

## Introduction

Bladder cancer is a common malignancy worldwide, with non-muscle invasive bladder carcinoma (NMIBC) accounting for approximately 70-80% of new diagnoses [1]. Despite its confinement to the mucosa or submucosa, NMIBC has a high tendency for recurrence and progression, necessitating effective intravesical therapy following transurethral resection of bladder tumor (TURBT) [2].

Bacillus Calmette-Guérin (BCG), an attenuated strain of *Mycobacterium bovis*, is an immunotherapy agent that triggers a localized immune response and enhances antitumor activity [3]. It remains the gold standard for intermediate- and high-risk NMIBC due to its demonstrated efficacy in reducing recurrence and delaying progression [4,5]. However, BCG treatment can cause both local and systemic side effects, including cystitis, hematuria, fever, and, rarely, life-threatening sepsis [6]. In addition, global shortages of BCG have disrupted treatment availability and led to increasing interest in alternative agents [7,8].

Mitomycin C (MMC) is a chemotherapeutic agent with direct cytotoxic effects on bladder tumor cells, acting through DNA cross-linking and inhibition of cell proliferation. It is commonly used in patients who are unsuitable for BCG or where BCG is not available [9]. While MMC effectively reduces recurrence, it is generally considered less effective than BCG in preventing disease progression, particularly in high-risk patients [3,10]. MMC is usually better tolerated, though it may cause chemical cystitis, bladder irritation, and treatment discontinuation in some cases [3].

Although many studies have compared BCG and MMC, their findings have often been inconsistent due to differences in study design, dosing schedules, and population characteristics [3,10]. Furthermore, updated comparative data from lower-middle-income countries like Pakistan remain limited, despite unique challenges such as BCG access issues, variability in treatment compliance, and differences in healthcare infrastructure [8,11].

This study aims to address these gaps by evaluating and comparing the efficacy and safety of intravesical BCG versus MMC in a contemporary Pakistani cohort of NMIBC patients, focusing on recurrence, progression, and adverse effects over a 24-month follow-up period. The findings are intended to provide practical insights for optimizing treatment in resource-constrained settings.

Research objective

The objective of this study is to compare the efficacy and safety of intravesical BCG and MMC in the treatment of NMIBC, with a focus on recurrence rates, disease progression, and adverse effects over a 24-month follow-up period in a tertiary care setting in Pakistan. This study aims to address evidence gaps and guide treatment decisions in resource-limited environments where BCG shortages and tolerance issues may influence therapeutic choices.

## Materials and methods

Ethical considerations

The study was approved by the Institutional Review Board, Khyber Medical College Peshawar (1017/DM/KMC), and all participants provided written informed consent. Given existing evidence favoring BCG in high-risk NMIBC, the study population was limited to patients with clinical equipoise (e.g., intermediate-risk or patients without prior BCG failure). The study protocol underwent ethical review to ensure that randomization did not compromise patient care.

Study design and setting

This comparative, prospective, randomized study was conducted at Khyber Teaching Hospital over a 24-month period from January 2023 to December 2024.

Patient selection criteria

Patients aged ≥18 years with histologically confirmed non-muscle-invasive bladder cancer (NMIBC), who had undergone complete TURBT, and were deemed eligible for intravesical therapy were included.

Exclusion criteria included muscle-invasive disease (≥T2), contraindications to BCG or MMC (e.g., immunosuppression and known allergy), active urinary tract infection, severe comorbidities likely to interfere with therapy or follow-up, and a history of prior systemic chemotherapy or radiation.

Sample size and sampling technique

The sample size was calculated based on prior studies reporting recurrence rates of ~25% for MMC and ~15% for BCG over a one- to two-year period. Using a two-sided chi-square test (α = 0.05, power = 80%), 130 patients per group were required. Accounting for a 10% loss to follow-up, the final sample size was 143 per group [12]. A convenience sampling method was used at a single tertiary center, which may limit external validity; however, consecutive patient recruitment was employed to reduce selection bias.

Randomization and blinding

Patients (n = 286) were randomly assigned to receive either BCG (n = 143) or MMC (n = 143) using computer-generated block randomization (block size = 4) to ensure balance across groups. Allocation was concealed using sealed opaque envelopes. While patients and treating physicians were not blinded due to differences in administration protocols and side effect profiles, outcome assessors (those evaluating cystoscopy and histopathology results) were blinded to treatment assignment to minimize assessment bias.

Intervention and dosage regimens

BCG (50 mg in 50 mL saline) and MMC (40 mg in 40 mL sterile water) were both administered intravesically once weekly for six weeks as induction. Maintenance therapy was administered per standard protocol. All regimens adhered to FDA-approved guidelines and institutional protocols.

Follow-up and outcome assessment

Patients were followed for a total of 24 months, with scheduled cystoscopy and urine cytology every three months during the first year and every six months in the second year. Tumor recurrence was defined as the reappearance of histologically confirmed urothelial carcinoma after initial complete resection, while progression was defined as advancement to muscle-invasive disease (stage ≥T2) or development of carcinoma in situ (CIS) with high-grade features. Any suspicious lesions identified during follow-up were biopsied to confirm recurrence or progression.

Adverse effects were systematically assessed at each follow-up visit using a standardized symptom checklist administered through structured patient interviews. In addition to clinical records and physician examination, adverse events were graded using the Common Terminology Criteria for Adverse Events (CTCAE) version 5.0 [13], ensuring consistency and objectivity in toxicity reporting. Each event was categorized according to CTCAE-defined criteria as Grade 1 (mild), Grade 2 (moderate), Grade 3 (severe), Grade 4 (life-threatening), or Grade 5 (death related to adverse events).

Patients were also instructed to maintain symptom diaries throughout the treatment and follow-up period. These diaries were used to document both local adverse effects (e.g., dysuria, urgency, frequency, and hematuria) and systemic effects (e.g., fever, malaise, and flu-like symptoms). Diaries were reviewed during clinic visits to supplement and verify patient-reported symptoms and CTCAE scoring.

Maintenance therapy was administered according to the Southwest Oncology Group schedule, consisting of three weekly instillations at three, six, and 12 months following induction. Adherence to maintenance therapy was closely monitored. All interruptions or discontinuations were recorded, with detailed documentation of reasons including adverse effects, patient noncompliance, or logistical issues, allowing assessment of the impact of tolerability on treatment continuation.

Statistical analysis

Data were analyzed using IBM SPSS Statistics for Windows, Version 20.0 (Released 2017; IBM Corp., Armonk, NY, USA). Descriptive statistics summarized baseline variables. Recurrence and progression were compared using chi-square tests, while continuous variables (e.g., time to recurrence) were analyzed using independent t-tests. Multivariable logistic regression was used to adjust for potential confounders (e.g., tumor grade, size, and multiplicity). Where appropriate, Bonferroni correction was applied to account for multiple comparisons. Analyses were performed on an intention-to-treat basis.

## Results

The research population’s baseline characteristics were similar for the MMC and BCG groups (Table 1). The BCG group’s mean age was 62.40 ± 8.70 years, whereas the MMC group’s was 61.80 ± 9.10 years. 71.33% and 68.53%, respectively, were men. Similarly, 40.56% of BCG patients and 38.46% of MMC patients reported currently smoking. In the MMC group, the tumor stage distribution was 43.36% Ta, 47.55% T1, and 9.09% CIS, while in the BCG group, it was 41.26% Ta, 50.35% T1, and 8.39% CIS. The BCG group had somewhat more high-grade tumors (65.73% vs. 63.64% in MMC). The rates of tumor multiplicity, size, and past recurrence were similar between groups; 40.56% of BCG patients had multiple tumors, compared to 38.46% of MMC patients, and 47.55% of BCG patients had prior recurrence, compared to 48.95% of MMC patients.

**Table 1:** Patient demographics and tumor characteristics at baseline BCG, Bacillus Calmette-Guérin; MMC, mitomycin C

Characteristic	Category	BCG group (n = 143)	MMC group (n = 143)
Age (years)	Mean ± SD	62.40 ± 8.70	61.80 ± 9.10
Gender	Male	102 (71.33%)	98 (68.53%)
	Female	41 (28.67%)	45 (31.47%)
Smoking history	Current smoker	58 (40.56%)	55 (38.46%)
	Former smoker	34 (23.78%)	36 (25.17%)
	Never smoked	51 (35.66%)	52 (36.36%)
Tumor stage	Ta	59 (41.26%)	62 (43.36%)
	T1	72 (50.35%)	68 (47.55%)
	CIS	12 (8.39%)	13 (9.09%)
Tumor grade	Low grade	49 (34.27%)	52 (36.36%)
	High grade	94 (65.73%)	91 (63.64%)
Multiplicity of tumors	Solitary	85 (59.44%)	88 (61.54%)
	Multiple	58 (40.56%)	55 (38.46%)
Tumor size, n (%)	<3 cm	77 (53.85%)	79 (55.24%)
	≥3 cm	66 (46.15%)	64 (44.76%)
Prior recurrence	n, %	68 (47.55%)	70 (48.95%)
Number of prior recurrences	0 (primary tumor)	75 (52.45%)	73 (50.98%)
	1	38 (26.57%)	40 (27.97%)
	≥2	30 (20.98%)	30 (20.98%)

**Figure 1:**
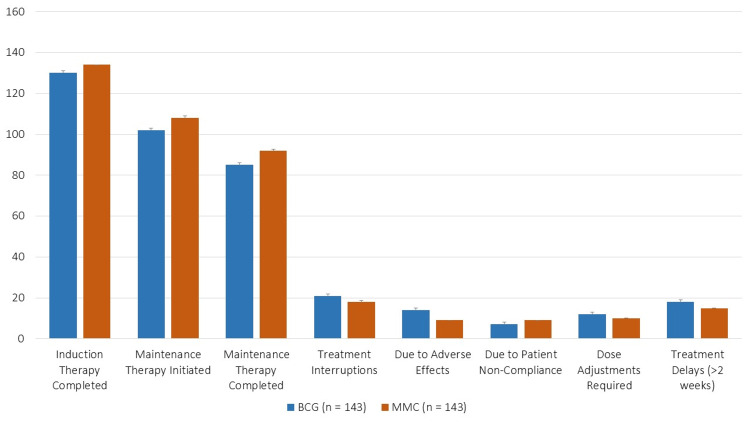
Treatment regimens and compliance BCG, Bacillus Calmette-Guérin; MMC, mitomycin C

Induction treatment completion rates were marginally higher in the MMC group (93.71%) than in the BCG group (90.91%) (Figure 1). Initiation and completion rates for maintenance treatment were 71.33% (BCG) vs. 75.52% (MMC) and 59.44% vs. 64.34%, respectively. Compared to 12.59% (MMC), 14.69% (BCG) had treatment interruptions, mostly as a result of patient noncompliance (4.90% vs. 6.29%) and adverse effects (9.79% vs. 6.29%). 8.39% (BCG) vs. 6.99% (MMC) needed dose modifications, and 12.59% vs. 10.49% had treatment delays longer than two weeks.

Table 2 shows that there was a statistically significant difference in the recurrence rates at six months (6.99% vs. 12.59%, p = 0.121), 12 months (13.29% vs. 22.38%, p = 0.037), and 24 months (23.78% vs. 34.27%, p = 0.043) between the BCG and MMC groups. 14.20 ± 4.80 months vs. 10.90 ± 5.30 months, p = 0.026). The BCG group had a substantially longer mean time to recurrence. Furthermore, the MMC group saw more multiple recurrences (15.38% vs. 8.39%, p = 0.049), suggesting that BCG is more effective in lowering the risk of recurrence.

**Table 2:** Recurrence rates in BCG vs. MMC groups Recurrences were analyzed using chi-square (χ²) tests. Mean comparisons are based on t-tests. ^*^ indicates statistical significance at p < 0.05. BCG, Bacillus Calmette-Guérin; MMC, mitomycin C

Outcome		BCG group (n = 143)	MMC group (n = 143)	p-Value	χ²	t-Value
Recurrence rates	Recurrence at six months	10 (6.99%)	18 (12.59%)	0.121	2.39	-
	Recurrence at 12 months	19 (13.29%)	32 (22.38%)	0.037^*^	4.36	-
	Recurrence at 24 months	34 (23.78%)	49 (34.27%)	0.043^*^	4.1	-
	Mean time to recurrence (months)	14.20 ± 4.80	10.90 ± 5.30	0.026^*^	-	2.24
	Multiple recurrences	12 (8.39%)	22 (15.38%)	0.049^*^	3.88	-
Progression rates	Progression at six months	5 (3.50%)	10 (6.99%)	0.189	1.72	-
	Progression at 12 months	9 (6.29%)	18 (12.59%)	0.048^*^	3.91	-
	Progression at 24 months	15 (10.49%)	29 (20.28%)	0.021^*^	5.36	-
	Progression to muscle-invasive disease (≥T2)	10 (6.99%)	22 (15.38%)	0.032^*^	4.61	-
	Progression to metastatic disease	3 (2.10%)	7 (4.90%)	0.198	1.65	-

**Figure 2:**
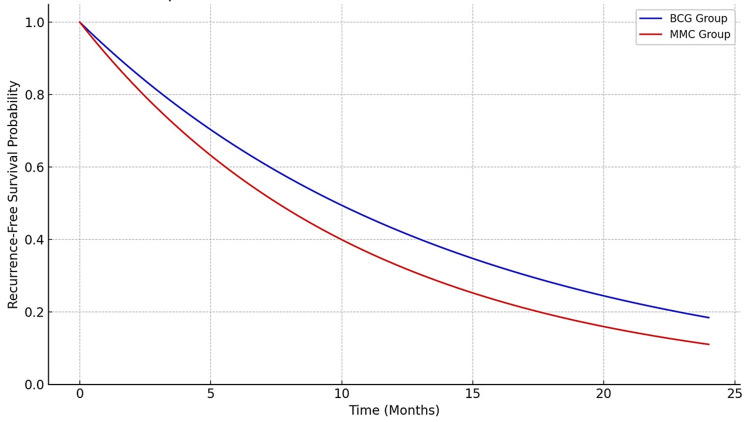
Kaplan-Meier analysis of time to recurrence over the 24-month follow-up period BCG, Bacillus Calmette-Guérin; MMC, mitomycin C

Kaplan-Meier analysis of time to recurrence over the 24-month follow-up period revealed a significantly higher recurrence-free survival in the BCG group compared to the MMC group (Figure 2). The BCG group demonstrated a more gradual decline in recurrence-free survival, with a longer mean time to recurrence (14.2 ± 4.8 months vs. 10.9 ± 5.3 months; p = 0.026). These results visually support the statistical findings that BCG is more effective at delaying recurrence in patients with NMIBC.

The BCG group saw less disease progression than the MMC group at six months (3.50% vs. 6.99%, p = 0.189), 12 months (6.29% vs. 12.59%, p = 0.048), and 24 months (10.49% vs. 20.28%, p = 0.021). These differences were statistically significant at subsequent time periods, as shown in Table 3. Better long-term disease control with BCG was indicated by the MMC group’s significantly higher progression to muscle-invasive disease (≥T2) (*15.38% vs. 6.99%, p = 0.032) and more frequent but not statistically significant progression to metastatic disease (4.90% vs. 2.10%, p = 0.198).

**Table 3:** Disease progression in BCG vs. MMC groups ^*^ indicates statistical significance at p < 0.05. BCG, Bacillus Calmette-Guérin; MMC, mitomycin C

Progression parameter	BCG group (n = 143)	MMC group (n = 143)	p-Value	χ²
Progression at six months	5 (3.50%)	10 (6.99%)	0.189	1.73
Progression at 12 months	9 (6.29%)	18 (12.59%)	0.048^*^	3.91
Progression at 24 months	15 (10.49%)	29 (20.28%)	0.021^*^	5.33
Progression to muscle-invasive disease ≥T2	10 (6.99%)	22 (15.38%)	0.032^*^	4.61
Progression to metastatic disease	3 (2.10%)	7 (4.90%)	0.198	1.65

The BCG group had somewhat more adverse effects (46.85% vs. 40.55%, p = 0.312), although they were not statistically significant (Table 4). Hematuria (15.38% vs. 12.58%, p = 0.529), chemical cystitis (26.57% vs. 31.46%, p = 0.371), and urine urgency/frequency (21.67% vs. 18.18%, p = 0.497) were among the local side effects that were similar between groups. Fatigue (11.88% vs. 6.99%, p = 0.183) and arthralgia (6.29% vs. 3.49%, p = 0.288) were more common but not statistically significant, whereas fever (13.28% vs. 4.19%, p = 0.012) and flu-like symptoms (16.78% vs. 5.59%, p = 0.004) were the most common systemic adverse effects associated with BCG. Although there was no discernible difference, the BCG group had more severe side effects, such as BCG sepsis (2.09% vs. 0.0%, p = 0.247) and therapy termination because of side effects (9.79% vs. 6.29%, p = 0.287).

**Table 4:** Adverse effects associated with BCG and MMC ^* ^indicates a statistically significant difference at p < 0.05. All p-values are derived from the chi-square test (χ²). BCG, Bacillus Calmette-Guérin; MMC, mitomycin C

Adverse effect		BCG group (n = 143)	MMC group (n = 143)	p-Value	χ²
Any adverse effect		67 (46.85%)	58 (40.55%)	0.312	1.03
Local adverse effects	Chemical cystitis	38 (26.57%)	45 (31.46%)		0.8
	Hematuria	22 (15.38%)	18 (12.58%)	0.529	0.4
	Urinary frequency/urgency	31 (21.67%)	26 (18.18%)	0.497	0.46
Systemic adverse effects	Fever	19 (13.28%)	6 (4.19%)	0.012^*^	6.34
	Flu-like symptoms	24 (16.78%)	8 (5.59%)	0.004^*^	8.33
	Fatigue	17 (11.88%)	10 (6.99%)	0.183	1.78
	Arthralgia (joint pain)	9 (6.29%)	5 (3.49%)	0.288	1.13
Severe adverse effects	BCG sepsis	3 (2.09%)	0 (0.0%)	0.247	1.34
	Severe allergic reaction	1 (0.69%)	2 (1.39%)	0.568	0.33
	Treatment discontinuation due to adverse effects	14 (9.79%)	9 (6.29%)	0.287	1.13

According to Table 5, normal results from follow-up cystoscopy were seen in 68.53% of BCG patients compared to 59.44% of MMC patients (p = 0.102). Compared to 21.68% of MMC patients, 14.69% of BCG patients had suspicious lesions (p = 0.098). Progression to muscle-invasive disease (≥T2) was substantially greater in the MMC group (6.99% vs. 15.38%, p = 0.032), and biopsy-proven recurrence was significantly lower in the BCG group (23.78% vs. 34.27%, p = 0.043). While bladder mucosal inflammation was similar in both groups (31.47% vs. 36.36%, p = 0.412), CIS was detected in 3.50% of BCG patients vs. 8.39% of MMC patients (p = 0.091).

**Table 5:** Follow-up cystoscopy findings ^*^ indicates statistical significance at p < 0.05. BCG, Bacillus Calmette-Guérin; CIS, carcinoma in situ; MMC, mitomycin C

Cystoscopy findings	BCG group (n = 143)	MMC group (n = 143)	p-Value	χ²
Normal findings	98 (68.53%)	85 (59.44%)	0.102	2.68
Suspicious lesions	21 (14.69%)	31 (21.68%)	0.098	2.76
Biopsy-proven recurrence	34 (23.78%)	49 (34.27%)	0.043^*^	4.1
CIS	5 (3.50%)	12 (8.39%)	0.091	2.87
Progression to muscle-invasive disease ≥T2	10 (6.99%)	22 (15.38%)	0.032^*^	4.61
Bladder mucosal inflammation	45 (31.47%)	52 (36.36%)	0.412	0.67

## Discussion

Our study demonstrates that intravesical BCG therapy is significantly more effective than MMC in reducing both tumor recurrence and disease progression among patients with NMIBC. At 12 months, the recurrence rate in the BCG group was significantly lower than in the MMC group (13.29% vs. 22.38%, p = 0.037), and this trend persisted at 24 months (23.78% vs. 34.27%, p = 0.043). These findings align with previous studies reporting superior recurrence control with BCG compared to MMC [7,14].

BCG also proved more effective in reducing disease progression. After 24 months, only 10.49% of BCG-treated patients showed progression compared to 20.28% in the MMC group (p = 0.021), and progression to muscle-invasive disease (≥T2) was notably lower in the BCG group (6.99% vs. 15.38%, p = 0.032). These outcomes are consistent with prior reports highlighting BCG’s role in reducing advancement risk in NMIBC patients [7].

Additionally, the BCG group demonstrated a significantly longer mean time to recurrence (14.20 ± 4.80 months) than the MMC group (10.90 ± 5.30 months, p = 0.026), suggesting that BCG provides a more durable treatment response. This is in accordance with earlier research indicating that BCG therapy prolongs recurrence-free survival [15].

The superior efficacy of BCG may be attributed to its immunologic mechanism of action. BCG elicits a robust immune response by stimulating both innate and adaptive immunity, recruiting antigen-presenting cells, enhancing cytokine production, and activating T-cell-mediated cytotoxicity against residual tumor cells [16]. In contrast, MMC exerts a direct cytotoxic effect but lacks this prolonged immune modulation. Reflecting this efficacy, both the European Association of Urology and the American Urological Association continue to endorse BCG as the standard first-line therapy for intermediate- and high-risk NMIBC [17,18].

However, BCG treatment carries certain logistical and clinical challenges. It requires careful handling, trained personnel, longer instillation durations, and closer monitoring for systemic adverse events. MMC, while less effective in preventing progression, may be more practical in settings with limited resources or BCG shortages and may be preferred by some patients due to its comparatively milder adverse effect profile. Thus, treatment decisions should be individualized, balancing disease risk, patient preference, healthcare capacity, and drug availability.

Regarding treatment compliance, the MMC group had slightly higher induction (93.71% vs. 90.91%) and maintenance completion rates (64.34% vs. 59.44%) than the BCG group. This difference may partly reflect BCG’s higher adverse effect burden, which has been linked to greater discontinuation in prior studies [6]. In our cohort, adverse effects were more frequently reported in the BCG group (46.85%) than in the MMC group (40.55%), although the difference was not statistically significant (p = 0.312). Notably, systemic adverse effects such as fever (13.28% vs. 4.19%, p = 0.012) and flu-like symptoms (16.78% vs. 5.59%, p = 0.004) were significantly more common with BCG, consistent with findings from earlier research [19].

Cystoscopy findings further supported BCG’s superior oncological control. A higher proportion of BCG patients had normal cystoscopic findings (68.53% vs. 59.44%, p = 0.102), and biopsy-confirmed recurrence was significantly lower in the BCG group (23.78% vs. 34.27%, p = 0.043). These results are in line with other studies demonstrating long-term disease control with BCG therapy [20].

Strengths and limitations

This study’s strengths include its prospective design, balanced sample size, and a 24-month follow-up period, which together enabled a robust evaluation of both early and delayed recurrences and disease progression. The trial was conducted in a routine tertiary care setting, which supports the applicability of the findings to everyday clinical practice, especially in resource-constrained healthcare environments.

However, the study has several limitations. The single-center design may limit the external validity of the results. Despite random allocation, variations in maintenance therapy adherence and patient compliance may have influenced treatment outcomes. Adverse effects were primarily captured through self-reporting and medical chart reviews, which may have led to underreporting, particularly of systemic symptoms. Additionally, although 24 months represents a meaningful follow-up period, NMIBC is known for potential late recurrences and progression; therefore, extended follow-up is necessary to fully assess long-term efficacy and safety.

Future multicenter investigations with standardized adverse event reporting, longer follow-up durations, and risk-based stratification are recommended to validate and expand upon these findings.

## Conclusions

Over a 24-month follow-up period, our study demonstrates that intravesical BCG therapy is superior to MMC in reducing both recurrence and disease progression among patients with NMIBC. The significantly lower rates of tumor recurrence and progression, as well as the longer mean time to recurrence observed in the BCG group, reinforce its role as the standard of care for intermediate- and high-risk NMIBC. However, BCG was also associated with a higher incidence of systemic adverse effects and slightly lower treatment adherence, underscoring the need for proactive strategies to improve patient tolerance and compliance throughout therapy.

These findings support the continued use of BCG as the preferred first-line intravesical therapy in eligible NMIBC patients, in alignment with current clinical guidelines. The choice of treatment must consider individual risk factors, potential side effects, and institutional capabilities. In clinical practice, a patient-centered approach, accounting for tolerance, comorbidities, drug availability, and logistical factors, remains crucial to optimizing outcomes and ensuring the sustainability of BCG-based treatment strategies.
